# Impact of pathological complete response following neoadjuvant chemotherapy (gemcitabine, nab-paclitaxel, S-1) and radiation for borderline resectable pancreatic cancer: a case report and review of literature

**DOI:** 10.1186/s40792-022-01529-z

**Published:** 2022-09-14

**Authors:** Mitsuru Kinoshita, Sota Watanabe, Gaku Mizojiri, Akitada Sada, Hiroki Kai, Yasunori Masuike, Yoshinobu Nagasawa, Kentaro Maruyama, Kyowon Lee, Mai Ohata, Osamu Ishikawa, Hiroshi Oka

**Affiliations:** 1 Department of Surgery, Moriguchi-Keijinkai Hospital, Moriguchi, Japan; 2 Department of Pathology, Moriguchi-Keijinkai Hospital, Moriguchi, Japan

**Keywords:** Borderline resectable pancreatic cancer, Neoadjuvant chemoradiation therapy, Pathological complete response

## Abstract

**Background:**

Pancreatic cancer (PC) is a highly lethal malignancy, even if surgical resection is possible (median survival: < 30 months). The prognosis of borderline resectable pancreatic cancer (BR-PC) is even worse. There is no clear consensus on the optimal treatment strategy, including pre/postoperative therapy, for BR-PC. We report a patient with BR-PC who achieved clinical partial response with neoadjuvant chemoradiation therapy (NACRT) and underwent curative resection, resulting in pathological complete response (pCR).

**Case presentation:**

A 71-year-old man with jaundice and liver dysfunction was referred to our department because of a 48-mm hypo-vascular mass in the pancreatic head with obstruction of the pancreatic and bile ducts and infiltration of superior mesenteric vein and portal vein. The lesion was identified as atypical cells which suggested adenocarcinoma by biopsy, and he was administered NACRT: gemcitabine and nab-paclitaxel, following S-1 and intensity modulated radiation therapy. After reduction in the tumor size (clinical partial response), pancreaticoduodenectomy was performed, and pCR achieved. Postoperative adjuvant chemotherapy with S-1 was initially administered and the patient is currently alive with no recurrence as of 2 years after surgery.

**Conclusions:**

NACRT is a potentially useful treatment for BR-PC that may lead to pCR and help improve prognosis.

## Introduction

Pancreatic cancer (PC) is an intractable disease with a poor prognosis. Only 15–20% of patients are eligible for curative surgical resection at the time of diagnosis, and the 5-year survival rate is extremely low (~ 20%) [1]. Neoadjuvant chemotherapy (NAC) or neoadjuvant chemoradiation therapy (NACRT) for PC is currently being developed with the objective of improving the outcomes of these patients. NAC and NACRT are poised to become an important part of multidisciplinary treatment for PC [2, 3]. Generally, neoadjuvant therapy (NAT) is expected to cause tumor regression and help improve the chances of curative surgery, and even survival rate. However, similar to biliary tract cancer, PC is considered to be less sensitive to NAT than other cancer types due to its biological characteristics (such as, intra-tumor heterogeneity, progression, and differentiation) [4–6]. Evans et al. proposed the histological efficacy criteria (Evans classification) for PC in 1992, which are widely used in Japan, Europe, and the United States [7]. Clinically, this has been shown as a predictor of prognosis, and it is extremely rare to achieve a pathological complete response (pCR), especially in terms of therapeutic efficacy.

The concept of borderline resectable pancreatic cancer (BR-PC) was recently proposed and defined as a tumor with abutment, encasement, or occlusion of a major vessel such as the portal vein, superior mesenteric vein, abutment of superior mesenteric artery < 180°, and tumor with a high percentage of residual cancerous tissue remaining even after surgery [3]. BR-PC has a poorer prognosis than resectable PC (R-PC) and is associated with a higher rate of postoperative recurrence and metastasis [8]. Therefore, the approach to BR-PC and R-PC should be different, and there is no clear consensus on the appropriate treatment for BR-PC. Herein, we report a highly suggestive case of pCR (no viable tumor) after NACRT (gemcitabine, nab-paclitaxel, S-1, and radiation) for BR-PC and summarize the key findings of a review of pertinent literature.

## Case presentation

**Fig. 1:**
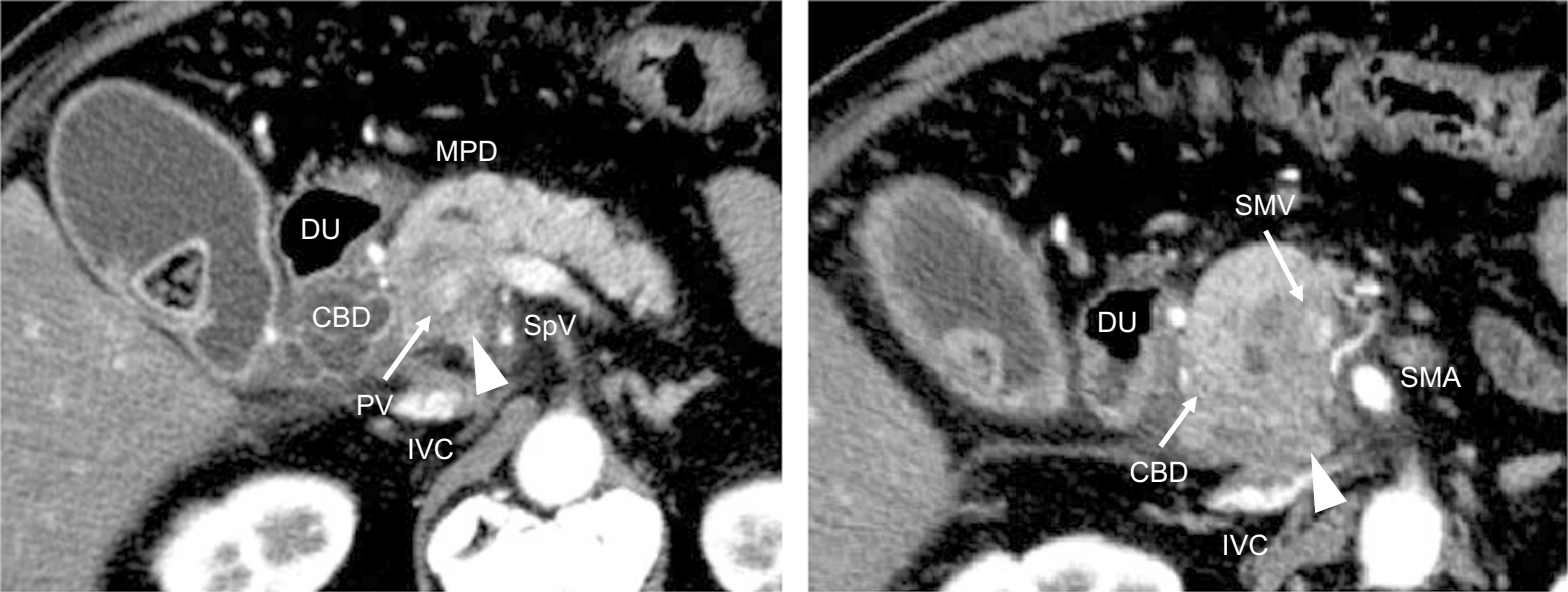
Preoperative enhanced computed tomography (CT). Abdominal axial CT shows a 48-mm hypo-vascular mass in the pancreatic head (arrowhead) involving the superior mesenteric vein and portal vein all around (arrow)

**Fig. 2:**
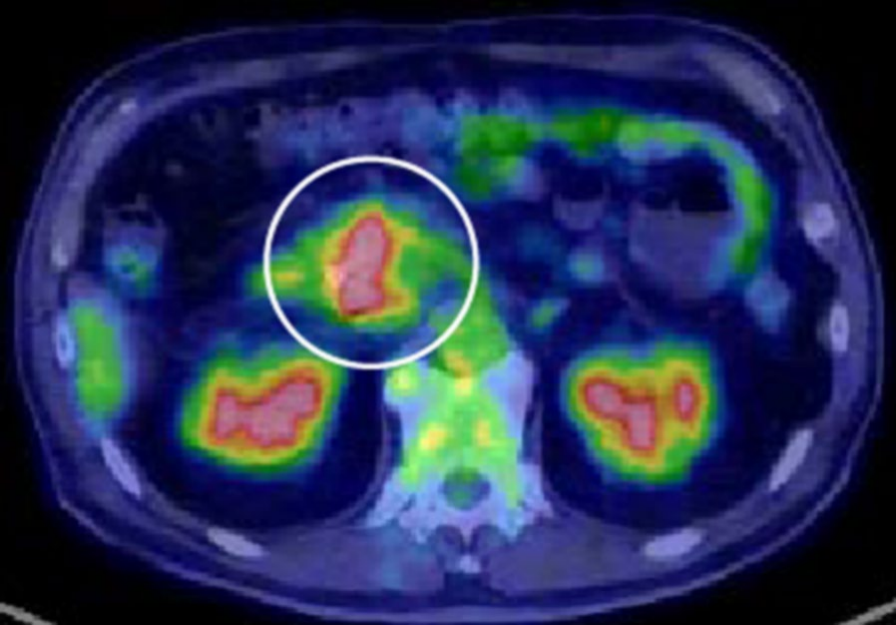
The 18F-fluorodeoxyglucose-PET (FDG-PET). FDG-PET CT showed abnormal accumulation in the pancreatic head (the maximum standardized uptake value [SUV] max = 9.5)

**Fig. 3:**
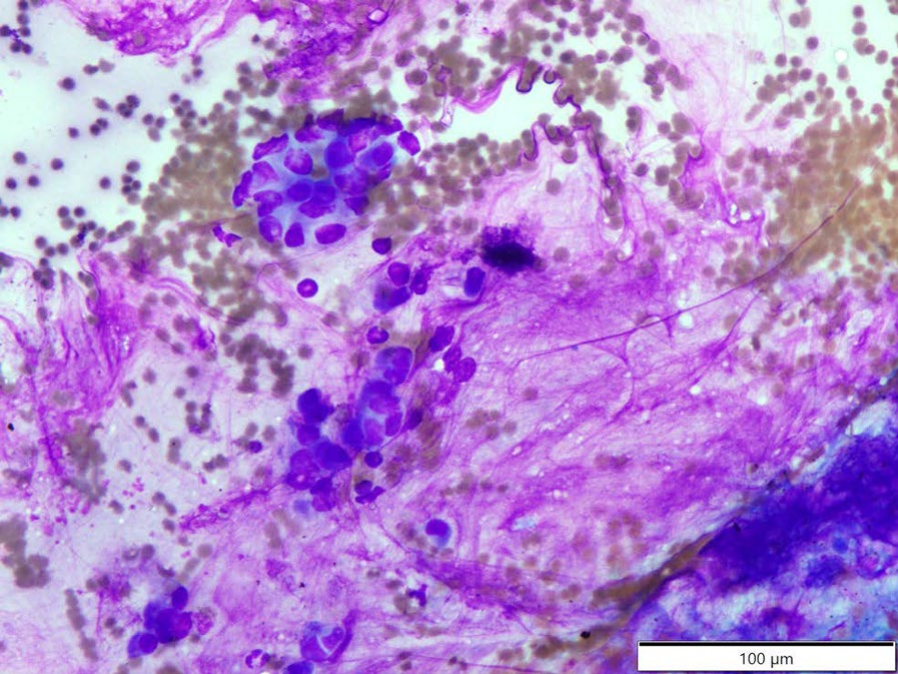
Histopathological findings. Endoscopic ultrasound-guided fine needle aspiration (EUS-FNA) rapid cytology showing the presence of some atypical cells which suggested adenocarcinoma cells (Giemsa stain, × 40)

**Fig. 4:**
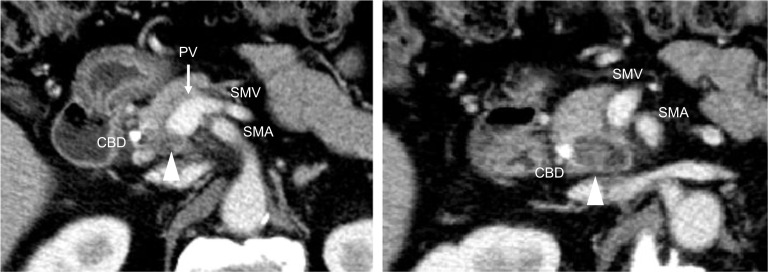
Enhanced computed tomography (CT) after neoadjuvant chemoradiation. Abdominal CT demonstrates reduction of tumor, 20 mm (arrowhead), with clinical partial response; the boundary with the superior mesenteric vein and portal vein (arrow) is clear

**Fig. 5:**
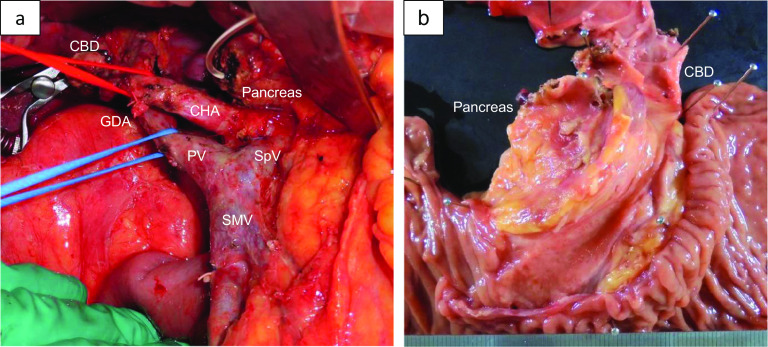
Operative photographs. Intraoperative photographs showing pancreaticoduodenectomy without resection and reconstruction of the portal vein (**a**). Resected specimen (**b**)

**Fig. 6:**
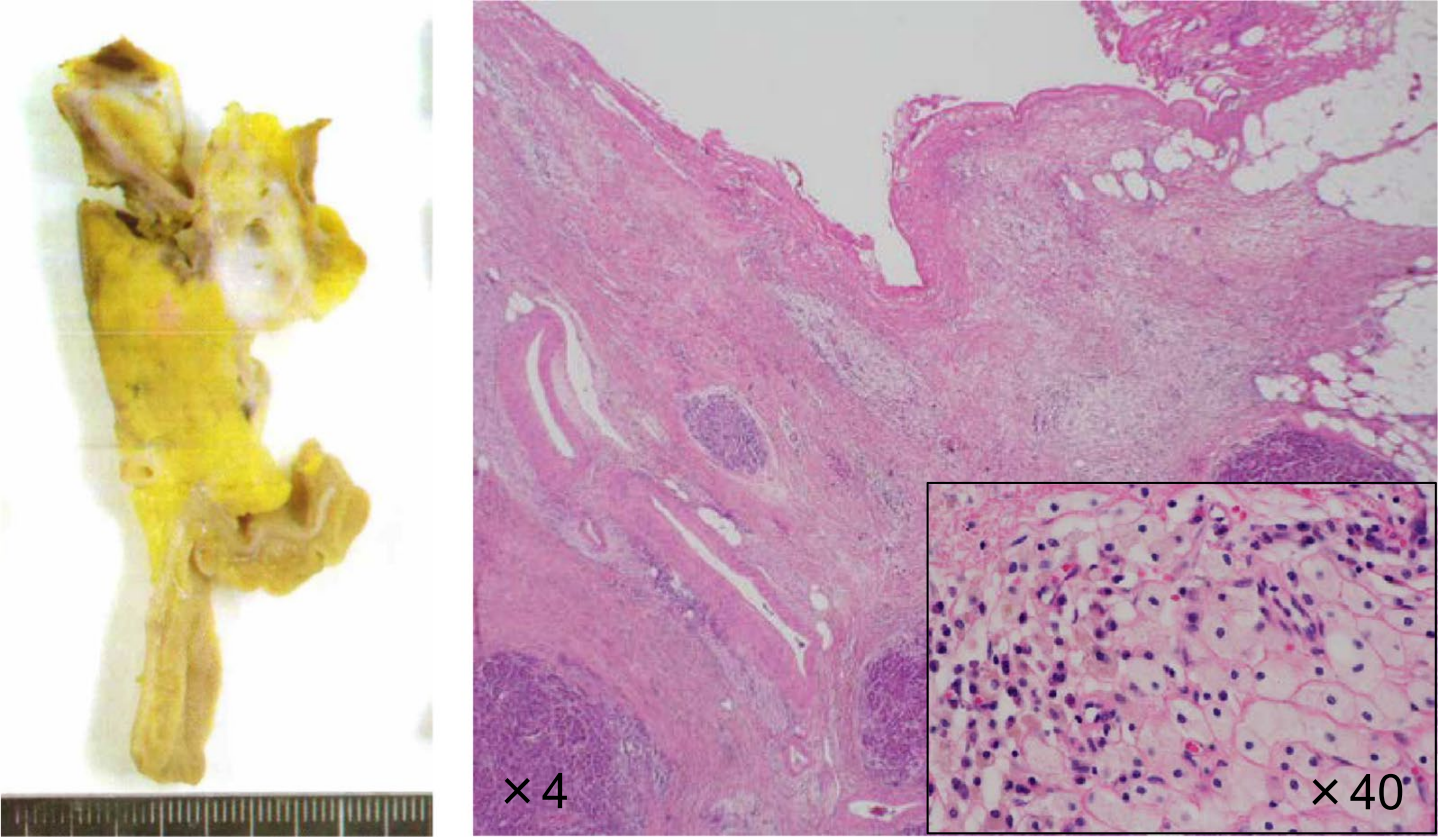
Histopathological findings. Histopathological findings reveal no residual adenocarcinoma. Fibrosis and scattered clusters of foamy cells are seen (magnification: × 4; × 40)

A 71-year-old man was referred to our hospital with jaundice and liver dysfunction. He had no significant past medical history or family history of malignancy. He was a non-smoker and consumed alcohol occasionally. His laboratory parameters were: total bilirubin 5.7 mg/dL, aspartate aminotransferase 103 IU/L, alanine amino transferase 133 IU/L, lactate dehydrogenase 290 IU/L, gamma-glutamyltransferase 1298 IU/L, and alkaline phosphatase 1410 IU/L. Tumor markers (CEA, CA19-9, DUPAN-2, and Span-1) were within their respective normal range. Abdominal enhanced computed tomography (CT) demonstrated a 48-mm hypo-vascular irregular mass in the pancreatic head, along with dilation of the main pancreatic duct and common bile duct (Fig. 1). The tumor had infiltrated the superior mesenteric vein (SMV) and portal vein (PV) all around (> 180°), but there was no obvious invasion of superior mesenteric artery. Also, there was no tumor presence in lymph nodes, liver, and peritoneum. The 18F-fluorodeoxyglucose-PET (FDG-PET) showed abnormal accumulation in the pancreatic head (the maximum standardized uptake value [SUV] max = 9.5) (Fig. 2). Endoscopic ultrasound-guided fine needle aspiration (EUS-FNA) cytology from the pancreatic head revealed the presence of atypical cells which suggested adenocarcinoma (Fig. 3). We established a diagnosis of BR-PC [Union for International Cancer Control (UICC), cT3 cN0 cM0 cStage IIA] without invasion of the celiac artery and superior mesenteric artery, and decided to administer neoadjuvant chemoradiation therapy (NACRT) with the approval of the patient, family, and our hospital’s institutional review board. The NACRT regime was as follows: induction chemotherapy, gemcitabine (GEM) (800 mg/m^2^) and nab-paclitaxel (nab-PTX) (100 mg/m^2^) were administered for a total of four courses (day 1, 15 in 28-day cycle). After four courses, S-1 oral administration (120 mg/day, irradiation day only) and intensity modulated radiation therapy (IMRT) (60 Gy/25 fractions) around the pancreatic head and retro peritoneum were administered. No ≥ grade 3 adverse events were observed, and the treatment was completed. After NACRT, abdominal CT demonstrated reduction in the size of PC to 20 mm with clear delineation of the boundary with PV and SMV (Fig. 4). According to Response Evaluation Criteria in Solid Tumors (RECIST) guidelines (version 1.1), the therapeutic effect was judged as partial response (PR) to NACRT and curative surgical resection was deemed possible. Six months after the definitive diagnosis of PC, and 4 weeks after the completion of NACRT, we performed subtotal stomach-preserving pancreaticoduodenectomy with lymph node dissection (Fig. 5). Intraoperative examination showed no signs of liver metastasis or peritoneal dissemination, and surgical resection was performed after confirming negative ascites cytology. PC can be exfoliated at the border with PV and SMV; also, cytology of the dissection between the tumor and PV was negative. Therefore, resection and reconstruction with PV was not performed. Histological examination showed highly fibrotic background pancreatic tissue with scattered clusters of foam cells, but no evidence of residual adenocarcinoma, confirming a pCR (Fig. 6). The lymph nodes were also negative for malignancy. During the postoperative course, the patient developed pancreatic fistula (International Study Group of Pancreatic Fistula grade A), but the condition improved with conservative treatment, and he was discharged one month after surgery. He was prescribed 6 months of S-1 oral administration as adjuvant chemotherapy and is currently alive without recurrence for 2 years after surgery.

## Discussion and conclusions

We report a patient with borderline resectable pancreatic cancer (BR-PC) who achieved a clinical partial response (PR) with neoadjuvant chemoradiation therapy (NACRT) and curative surgical resection was performed, resulting in pathological complete response (pCR).

Recently, neoadjuvant therapy (NAT) has been widely used in various types of cancer, especially, esophageal cancer [9]. NAT inhibits the mitotic potential and progression of tumor cells, minimizes the chances of viable tumor cells remaining at the time of surgical resection, reduces the risk of tumor spread, and improves curability [2, 3, 5]. In addition, sufficient preoperative compliance may achieve a high completion rate.

Patients with pancreatic cancer (PC) typically have a low completion and implementation rate of postoperative adjuvant therapy due to high invasiveness of surgical procedures and occurrence of complications. Therefore, development of NAT with higher efficacy and feasibility is in progress.

In the Pep02/JSAP05 trial, neoadjuvant chemotherapy (NAC) with gemcitabine (GEM) and S-1 achieved a significantly better overall survival (OS) than upfront surgery [36.7 vs. 26.6 months; hazard ratio (HR) = 0.72, *p *= 0.015]. It was a pivotal trial that demonstrated the benefit of NAC for PC [10]. Based on these results, NAC for PC was added to the guidelines of the Japan Pancreas Society. In addition, RCTs conducted in Europe and the United States have shown the efficacy of NAC for PC. Moreover, the National Comprehensive Cancer Network (NCCN) guidelines allow the use of NAC for PC in clinical trials [11]. Based on the resectability status, the NCCN guidelines classify PC into resectable PC (R-PC), BR-PC, and unresectable PC (UR-PC). R-PC refers to PC that is amenable to radical surgical resection. BR-PC is PC in which there is a high possibility of pathologically residual cancer if treated only by surgery, while UR-PC refers to PC with invasion of major vessels or distant metastases. In the study by Yamada et al., the OS patients with R-PC, BR-PC with portal vein (PV) invasion, BR-PC with arterial invasion, and UR-PC was 34.2, 17.3, 14.3, and 15.8 months, respectively [8]. The OS of patients with BR-PC was significantly worse than that of patients with R-PC (*p* < 0.01). Therefore, more aggressive NAT is required to suppress the tumor progression, and improve negative resection margin rates.

The landmark trial by Jang et al. [12] revealed the superiority of GEM-based NACRT over upfront surgery for BR-PC (2-year OS and median survival: 40.7%, 21 months vs. 26.1%, 12 months; HR = 1.50, *p* = 0.028. R0 resection: 51.8 vs. 26.1%, *p* = 0.004) [12]. In the PREOPANC-1 trial, sub-group analysis limited to BR-PC showed favorable outcomes of GEM-based NACRT compared to upfront surgery (median OS: 17.6 vs. 13.2 months, HR = 0.62, p = 0.029. R0 resection: 79 vs. 13%, *p* < 0.001) [13]. These RCTs suggest that NACRT may be useful in the treatment for BR-PC. However, the optimal regimen including the duration of NAC/NACRT is currently being explored. In Japan, in addition to GEM and S-1, FOLFIRINOX (5-fluorouracil, oxaliplatin, irinotecan, and leucovorin) or GEM + nab-paclitaxel (albumin-coated formulation of paclitaxel) (Nab-PTX), which have shown promising results in UR-PC with metastasis, are often administered, and radiotherapy is also routinely administered in some institutions [14, 15]. The key consideration for NAT for BR-PC is to increase the possibility of R0 resection and eliminate micro-metastases, which may improve the prognosis.

At our institution, we have been actively running NACRT for BR-PC, referring to the Osaka International Cancer Institute regimen, which entails the addition of radiation to Gem-based chemotherapy [2, 3]. Depending on the patient’s tolerability, NACRT is being carried out as a part of clinical research, using S-1, which has a synergistic effect in increasing the tumor radiosensitivity in addition to the potent anti-tumor effect of GEM and Nab-PTX. PC typically shows low radiosensitivity, and it is not easy to administer sufficient dose by conventional radiotherapy due to the proximity of important organs such as the gastrointestinal tract. Therefore, IMRT, which enables high-precision delivery of appropriate dose to the target, and minimizes the exposure of healthy tissues, has been performed in recent years. At present, we have achieved high R0 resection rate, although this may be influenced to a certain extent by selection bias. In the present case, the tumor showed clinical PR, and curative surgery was possible because there was clear delineation of the boundary between the tumor and PV/the superior mesenteric vein. On intraoperative examination, there was no hardening or edema of the pancreatic tissue; thus, the NACRT did not enhance the complexity of surgery. This case provided valuable experience as the marked efficacy of NACRT enabled R0 resection, leading to pCR.

We systematically reviewed the pertinent literature published in English language using PubMed and Google Scholar (reference period: 2005 to 2020). The keywords used for database search were: “pathological complete response”, “preoperative/neoadjuvant chemo radiation”, and “borderline resectable pancreatic cancer”. We examined 16 studies that reported pCR rates following NACRT for BR-PC and locally advanced pancreatic cancer (Table 1) [12, 16, 17]. In most cases, the NACRT regimens were based on GEM, a key drug in PC, and FOLFIRINOX, and the total radiation dose ranged from 30 to 60 Gy. There were no previous reports of pCR achieved with GEM + Nab-PTX + S-1/radiation (IMRT). The rate of pCR ranged from 4–25% which was higher than that reported for NAC (3–11%) [17]. Although most of these studies did not perform detailed prognostic evaluation, many of the patients who achieved pCR were alive with no evidence of disease, but some patients developed metastases or recurrence.

**Table 1:** pCR cases after neoadjuvant chemoradiation therapy for borderline resectable and locally advanced pancreatic cancer (2005–2020)

Case	Authors	Journal	Year	pCR rate	Assessment	Chemotherapy	Radiation dose	Status Alive/dead
1	Katz et al	J Am Coll Surg	2008	4/66 (6.1%)	BR	5-FU or PTX or GEM or Cape	30 or 50.4 Gy	2 (NED)/2 (1 DOD, 1 DOA)
2	Rajagopalan et al	Radiat Oncol	2013	3/12 (25%)	BR + LA	GEM + Cape or FFN	36 Gy (SBRT)	NR
3	Rose et al	Ann Surg Oncol	2014	3/31 (9.7%)	BR	GD	50.4 Gy	NR
4	Pietrasz et al	Ann Surg Oncol	2015	12/80 (15%)	BR + LA	FFN	54 Gy	NR
5	Hirata et al	Radiother Oncol	2015	6/157 (4%)	BR + LA	GEM	50 Gy	NR
6	Katz et al	JAMA Surg	2016	2/22 (13%)	BR	FFN + Cape	50.4 Gy	NR
7	Chuong et al	J Gastrointest Oncol	2016	4/36 (11.1%)	BR	GTX	30–40 Gy (SBRT)	4 (NED)/0
8	Rashid et al	Ann Surg Oncol	2016	10/55 (18.2%)	BR	GTX	30–40 Gy (SBRT)	NR
9	Mellon et al	Acta Oncol	2017	6/81 (7.4%)	BR	GEM or GTX or GnP or FFN or others	30–50 Gy (SBRT)	6 (5 NED, 1 AWD; liver)/ 0
10	Hashemi-Sadraei et al	Am J Clin Oncol	2018	5/53 (9.4%)	BR	GEM or GEM + SFB or GEM + ERL or GnP or FFN	45–54 Gy	3 (2 NED, 1 AWD; unknown)/2 (1 DOD, 1 DOA)
11	Takahashi et al	Pancreas	2018	3/24 (12.5%)	BR	GnP	60 Gy	NR
12	Jang et al	Ann Surg	2018	2/17 (11.8%)	BR	GEM	54 Gy	NR
13	He et al	Ann Surg	2018	19/186 (10.2%)	BR + LA	GnP or GX or FFX	NR (SBRT or conventional)	8 (NED)/NE; 2 recurrence, 3 liver, 1 lung, 3 multiple
14	Blair et al	Surgery	2018	14/168 (8.3%)	BR + LA	5-FU or GEM or Cape or FFX or others	33 Gy (SBRT) or 45–54 Gy (CRT)	NR
15	Lewis et al	J Gastrointest Oncol	2019	2/30 (6.7%)	BR + LA	GEM	45–57 Gy (IMRT)	NR
16	Neyaz et al	Histopathology	2020	15/92 (16.3%)	BR + LA	FFX	50.4 Gy	NR

*BR* borderline resectable, *LA* locally advanced, *5-FU* 5-fluorouracil, *GEM* gemcitabine, *Cape* capecitabine, *GX* gemcitabine and capecitabine, *GTX* gemcitabine, docetaxel, and capecitabine, *FFN* FOLFIRINOX, *GnP* gemcitabine and nab- paclitaxel, *SFB* sorafenib, *ERL* erlotinib, *NED* no evidence of disease, *DOA* death of another disease, *NED* no evidence of disease, *AWD* alive with disease, *DOD* death of disease, *NA* not reported

On the other hand, Basem Azab et al. reported that among 2093 patients who underwent NAT for PC, 44 patients (2.1%) achieved pCR; in addition, NACRT group showed a significantly higher pCR rate than NAC (2.5 vs. 1.1%, *p* = 0.049) [18]. However, NACRT was an independent predictor for pCR, but not an independent prognostic factor for OS, although patients with pCR had better OS than non-CR (41 vs. 19 months, *p* = 0.03). Nonetheless, the study population included patients with stage I–III PC, and the results may not be entirely extrapolatable to more advanced BR-PC. Recent in vivo/vitro studies have reported that radiation induces hepatocyte growth factor (HGF) receptor, c-met expression, and matrix metalloproteinase (MMP)-2 activity in pancreatic cancer cells, promoting amplification of malignancy, especially, distant metastasis [19, 20]. Radiation is considered to be excellent for local control, but may not be suitable for controlling distant metastases.

In this case, tumor markers including CA19-9 were within their respective normal range, and Motoi et al. reported that the prognosis for patients with preoperative CA19-9 non-elevation was better than that for those with elevation. However, since this is a BR-PC case, we cannot be optimistic [21].

In conclusion, NACRT is a useful treatment strategy for BR-PC that may lead to pCR and help improve prognosis. However, postoperative recurrence and metastasis may occur even after achieving pCR, and therefore, meticulous and regular follow-up of these patients is necessary. There is no clear consensus on the choice between NACRT and NAC for BR-PC; therefore, large-scale clinical trials are required to determine the optimal treatment strategy.

## Data Availability

The data supporting the findings of this study are available within the article.

## Abbreviations

PC: Pancreatic cancer
BR-PC: Borderline resectable pancreatic cancer
R-PC: Resectable pancreatic cancer
UR-PC: Unresectable pancreatic cancer
NAT: Neoadjuvant therapy
NAC: Neoadjuvant chemotherapy
NACRT: Neoadjuvant chemo radiation therapy
pCR: Pathological complete response
PR: Partial response
RECIST: Response Evaluation Criteria in Solid Tumors
CT: Computed tomography
EUS-FNA: Endoscopic ultrasound-guided fine needle aspiration
SMV: Superior mesenteric vein
PV: Portal vein
GEM: Gemcitabine
PTX: Paclitaxel
IMRT: Intensity modulated radiation therapy
OS: Overall survival
HR: Hazard ratio
NCCN: National Comprehensive Cancer Network
HGF: Hepatocyte growth factor
MMP: Matrix metalloproteinase
